# 离子交换色谱-串联质谱法快速测定茶叶中氯酸盐和高氯酸盐

**DOI:** 10.3724/SP.J.1123.2023.10026

**Published:** 2024-09-08

**Authors:** Mingli YE, Guohua ZHAO, Yong WANG, Jirun LIN, Wenxin LIU, Jie LU, Yonggang ZHAO, Ban CAO

**Affiliations:** 1.浙江树人学院生物与环境工程学院,浙江杭州 310015; 1. College of Biological and Environmental Engineering, Zhejiang Shuren University, Hangzhou 310015, China; 2.实朴检测技术(上海)股份有限公司, 上海 201203; 2. SEP Analytical Services (Shanghai) Co., Ltd., Shanghai 201203, China; 3.东南大学化学化工学院,江苏南京 211189; 3. School of Chemistry and Chemical Engineering, Southeast University, Nanjing 211189, China; 4.南京市公安局刑事科学技术研究所,江苏南京 210001; 4. Institute of Forensic Science and Technology of Nanjing Public Security Bureau, Nanjing 210001, China; 5.浙江省地质院,浙江杭州 310015; 5. Zhejiang Institute of Geosciences, Hangzhou 310015, China

**Keywords:** 离子交换色谱-串联质谱, 氯酸盐, 高氯酸盐, 茶叶, ion exchange chromatography-tandem mass spectrometry (IEC-MS/MS), chlorate, perchlorate, tea

## Abstract

目前,氯酸盐和高氯酸盐作为新型持久性环境污染物受到了广泛关注。茶树在我国有大面积种植,而在受高氯酸盐污染的土壤中,其含有的氯酸盐和高氯酸盐可在茶树中富集,鉴于氯酸盐和高氯酸盐对人体具有的潜在健康风险,因此亟需建立一种茶叶中氯酸盐和高氯酸盐残留量的快速检测方法。本文建立了茶叶中氯酸盐和高氯酸盐残留量的离子交换色谱-串联质谱检测方法。本研究优选Oasis PRiME HLB SPE柱对样品提取液进行净化,使用AceChrom Hybri-A (150 mm×2.1 mm, 5.0 μm)作为离子交换色谱柱,以100 mmol/L乙酸铵-乙腈(40∶60, v/v)作为流动相,在电喷雾负离子和MRM模式下,对目标物实现快速、准确的定性分析,并采用内标法定量。研究结果表明:当氯酸盐与高氯酸盐分别在2.00~200 μg/L和1.00~100 μg/L范围内时,方法的线性关系良好(*r*^2^>0.9990);在低、中、高3个加标水平下,氯酸盐和高氯酸盐平均回收率为88.54%~97.25%,方法的检出限分别为12.0 μg/kg和8.0 μg/kg,方法的定量限分别为40.0 μg/kg和26.6 μg/kg。该方法简单、快速、灵敏、准确,能够满足大批量茶叶样品中氯酸盐和高氯酸盐的快速筛查与定量分析。

## Abstract (en)

Ion exchange chromatography-tandem mass spectrometry (IEC-MS/MS) has recently become the preferred method for detecting ionic substances in tea. In this study, an IEC-MS/MS method was developed for the rapid determination of chlorate and perchlorate residues in tea samples. The optimal sample extraction process, pretreatment column, and chromatographic and mass spectrometric conditions were systematically investigated. In the optimal process, the tea samples were ultrasonically extracted with methanol-water (13∶7, v/v), and a PRiME HLB SPE column was used to purify the sample extract. An AceChrom Hybri-A IEC column (150 mm×2.1 mm, 5.0 μm) was used for separation, and 100 mmol/L ammonium acetate-acetonitrile (40∶60, v/v) was used as the mobile phase for isocratic elution. The flow rate was 0.3 mL/min, the column temperature was 40 ℃, and the injection volume was 5.0 μL. The mass spectrometric data were collected in negative electrospray ionization mode combined with multiple reaction monitoring (MRM) mode to achieve the rapid and accurate separation and qualitative analysis of the desired chemical components. Quantification was performed using the internal standard (IS) method. The measurement results showed a good linear relationship when the mass concentrations of chlorate and perchlorate were between 2.00-200 and 1.00-100 μg/L, respectively, with correlation coefficients (*r*^2^) greater than 0.9990. The average recoveries of chlorate and perchlorate at three spiked levels of low, medium, and high ranged from 88.54% to 97.25% with relative standard deviations (RSDs, *n*=7) of 3.2%-5.2%. The limits of detection for chlorate and perchlorate were 12.0 and 8.0 μg/kg, respectively, while the limits of quantification were 40.0 and 26.6 μg/kg, respectively. The results of tests conducted to assess the linearity, specificity, accuracy, precision, and applicability of the method to the analysis of chlorate and perchlorate in 15 tea samples collected from a local market demonstrated its validity for the routine analysis of tea samples. The proposed method is simple, rapid, sensitive, and accurate, and can meet requirements for the rapid screening and quantitative analysis of residual trace chlorate and perchlorate in large quantities of tea samples.

目前,氯酸盐(Cl ${O_{3}}^{-}$ )和高氯酸盐(Cl ${O_{4}}^{-}$ )作为新型持久性环境污染物受到了广泛关注。工业、医学、农业和海水提溴等领域多采用二氧化氯、次氯酸盐与液氯对水进行消毒,然而该过程中会形成副产物氯酸盐^[1,2]^,该污染物能破坏血红细胞,导致人体罹患高铁血红蛋白血症和溶血性贫血^[3]^。高氯酸盐因具有良好的抗氧化能力和耐腐蚀特点,被广泛应用于航天、军事、烟草、制鞋和涂料行业^[4,5]^。然而,高氯酸盐作为一种强烈的碘吸收抑制剂,在低碘水平的敏感人群中会增加神经发育受损的风险^[6⇓-8]^。此外,长期摄入氯酸盐和高氯酸盐会干扰甲状腺的正常功能,导致甲状腺功能紊乱,甚至增加甲状腺癌等甲状腺疾病的发病几率^[9⇓-11]^。因此,减少氯酸盐与高氯酸盐暴露是公共卫生的优先事项。鉴于氯酸盐和高氯酸盐对人体健康具有潜在风险,许多国家、地区及机构都已采取各种措施加以控制^[12⇓-14]^。目前,我国尚没有食品中氯酸盐与高氯酸盐的相关限量标准,因此亟需建立一种食品中氯酸盐和高氯酸盐残留量的检测方法。

茶树作为一种极具发展潜力的经济作物,已在我国大面积种植。研究表明种植茶树的土壤一旦受到高氯酸盐的污染,土壤中含有的氯酸盐和高氯酸盐最终会在茶树中富集^[15]^。因此开展茶叶样品中氯酸盐和高氯酸盐的分析检测研究具有重要的现实意义。

近年来,用于检测食品中氯酸盐和高氯酸盐的分析方法主要包括分光光度法、液相色谱-质谱联用法(LC-MS/MS)^[16,17]^、离子色谱法(IC)^[18,19]^及离子色谱-三重四极杆质谱联用法(IC-MS/MS)^[20⇓-22]^等。其中,IC-MS/MS因检测灵敏度高、选择性好被广泛用于食品中痕量氯酸盐、高氯酸盐的分析测定。2009年,Mari等^[23]^采用IC-MS/MS对106种瓶装饮料中的氯酸盐和高氯酸盐进行了测定,用于评估瓶装饮料中氯酸盐和高氯酸盐的暴露风险。结果表明:所检测的饮料中均含有这两种物质,部分饮料中的含量超过了最低标准限值。2021年,Zhang等^[24]^采用IC-MS/MS对现煮咖啡中氯酸盐和高氯酸盐进行了测定,结果表明氯酸盐和高氯酸盐在咖啡饮品中普遍存在。同年,张昊等^[25]^建立了一种非抑制性IC-MS/MS方法,用于婴幼儿配方奶粉中氯酸盐和高氯酸盐的测定,方法的检出限为4.8 μg/kg和1.6 μg/kg。

上述食品样品中氯酸盐与高氯酸盐的前处理方法多采用传统的固相萃取技术(活化-上样-淋洗-洗脱),虽然可以获得良好的方法回收率与精密度,但是耗时长,操作步骤繁琐。本文在传统固相萃取技术的基础上,采用通过型固相萃取技术(保留样品共萃物,目标分析物直接随流出液流出),对茶叶样品中的氯酸盐和高氯酸盐进行快速净化,极大提高了固相萃取效率,较传统的固相萃取更加快速,缩短了样品前处理时间,进而利于大批量茶叶样品的分析检测。

## 1 实验部分

### 1.1 仪器、试剂与材料

配有SM-FTN液体自动进样器、I-CLASS双柱塞循环往复泵和CH-A柱温箱的I-CLASS PLUS液相色谱仪,XEVO TQS型三重四极杆质谱仪,MassLynx色谱-质谱数据管理系统,Oasis PRiME HLB亲水亲脂固相萃取柱(美国Waters公司); AceChrom Hybri-A色谱柱(150 mm×2.1 mm, 5.0 μm,固定相功能基团为季铵基阴离子交换基团)(杭州和谱新材料有限公司); TGL-20bR型离心机(上海安亭科学仪器厂)。

氯酸盐标准储备溶液(1000 mg/L)、高氯酸盐标准储备溶液(1000 mg/L)、甲酸(色谱纯)、乙酸铵(LC-MS级)、乙腈(色谱纯) (上海安谱实验科技有限公司);氯酸盐-^18^O_3_标准溶液(200 mg/L, EURL-SRM公司);高氯酸盐-^18^O_4_标准溶液(100 mg/L, BePure公司)。茶叶样品为市售。

### 1.2 标准溶液配制

分别移取20 μL氯酸盐和10 μL高氯酸盐标准储备溶液于10.0 mL容量瓶中,用水稀释至刻度,摇匀,得到氯酸盐与高氯酸盐质量浓度分别为2.00 μg/mL和1.00 μg/mL的混合标准溶液。

准确移取50 μL氯酸盐-^18^O_3_标准溶液及100 μL高氯酸盐-^18^O_4_标准溶液至10.0 mL容量瓶中,用水稀释并定容,配制氯酸盐-^18^O_3_、高氯酸盐-^18^O_4_质量浓度为1.00 μg/mL的混合同位素内标溶液。

分别移取一定量的混合标准溶液与混合同位素内标溶液配制氯酸盐和高氯酸盐系列标准工作液,用乙酸铵-乙腈溶液(40∶60, v/v)稀释至10.0 mL,其中:氯酸盐的质量浓度分别为2.00、5.00、10.0、20.0、50.0、100与200 μg/L;高氯酸盐的质量浓度分别为1.00、2.50、5.00、10.0、25.0、50.0与100 μg/L;氯酸盐-^18^O_3_与高氯酸盐-^18^O_4_的质量浓度均为10.0 ng/mL。

### 1.3 实验方法

#### 1.3.1 样品制备

取50 g茶叶,经粉碎机粉碎后过40目筛,过筛后均分成试样和留样,分别装封,用封口膜密封并标记后置于常温避光处保存。

#### 1.3.2 样品提取

称取1.00 g(精确至0.001 g)茶叶样品,置于塑料聚乙烯离心管(50 mL)中,准确移取200 μL 1000 ng/mL的混合同位素内标液,加入7.0 mL水,涡旋5 min,加入13.0 mL甲醇,超声提取30 min,以10000 r/min离心10 min,移取上清液,待用。

#### 1.3.3 样品净化

准确移取3.0 mL茶叶提取液,经Oasis PRiME HLB柱净化,再经0.22 μm滤膜过滤,弃去前面先流出的1 mL流出液,收集后续液,供IC-MS/MS测定。

### 1.4 色谱-质谱分析条件

色谱柱为AceChrom Hybri-A (150 mm×2.1 mm, 5.0 μm);流动相:0.1 mol/L乙酸铵水溶液-乙腈(40∶60, v/v),等度洗脱;进样量:5 μL;柱温为40 ℃;流速为0.3 mL/min。

离子源:电喷雾电离源;离子化模式:负电离模式;离子源温度:150 ℃;脱溶剂气温度:500 ℃;毛细管电压:0.5 kV;脱溶剂气流量:1000 L/h;检测模式:多反应监测(MRM);锥孔气流量:150 L/h;碰撞气与脱溶剂气:N_2_。目标分析物的离子对信息、碰撞电压及锥孔电压等质谱条件见表1。

**表1:** 氯酸盐和高氯酸盐的质谱参数

Analyte	Precursor ion (*m/z*)	Product ion (*m/z*)	Declustering potential/V	Collision energy/eV
Cl ${O_{3}}^{-}$	82.97	67.01^*^	74	12
	84.97	68.95	74	14
Cl ${O_{4}}^{-}$	98.90	82.99^*^	74	18
	100.90	84.98	74	20
Cl ${O_{3}}^{-}$ -^18^O_3_	88.97	71.01^*^	76	14
Cl ${O_{4}}^{-}$ -^18^O_4_	106.97	88.98^*^	72	22
		71.01	72	28

* Quantitative ion.

## 2 结果与讨论

### 2.1 净化流程优化

样品前处理在分析过程中占据着较大的比重,因此为了探究固相萃取小柱的种类对氯酸盐和高氯酸盐检测的影响,本研究对比了PRiME HLB、氧化铝和C18 3种固相萃取小柱。结果如表2所示,3种固相萃取柱对高氯酸盐的萃取效果无显著差别,但是对于氯酸盐的萃取效果有显著差别。PRiME HLB与氧化铝固相萃取小柱对氯酸盐的回收率高于C18固相萃取小柱,且PRiME HLB固相萃取小柱对氯酸盐的回收率高于氧化铝固相萃取小柱,因此实验选择PRiME HLB小柱对茶叶样品中的氯酸盐和高氯酸盐进行净化处理,其加标回收率为86.21%~96.72%。

**表2:** 不同种类SPE柱对目标分析物的萃取效率

Analyte	Spiked level/ (μg/kg)	Recoveries/%		
		PRiME HLB SPE	Al_2_O_3_ SPE	C18 SPE
Cl ${O_{3}}^{-}$	80	92.36	78.58	51.86
	800	86.21	82.54	62.85
	3200	90.17	88.85	57.25
Cl ${O_{4}}^{-}$	40	89.12	86.85	89.57
	400	93.17	92.68	91.85
	1600	96.72	89.78	93.51

PRiME HLB小柱具有极强的不可逆吸附性,当吸附饱和时,其对目标分析物的回收率趋于稳定。本研究以1 mL为单位收集了前3 mL的流出液,测定流出液中目标分析物的绝对响应值。测试结果表明,第1 mL流出液的绝对响应值明显低于第2 mL与第3 mL流出液,该现象可能是由于该SPE小柱对目标分析物有微弱的吸附作用,待其达到吸附饱和之后,流出液中目标分析物的浓度不再变化。鉴于此,本研究弃去第1 mL的流出液,并选择收集后续流出液进行测试,以此获得更高的方法精密度。

### 2.2 色谱流动相的优化

本研究重点考察了不同比例的0.1 mol/L乙酸铵水溶液-乙腈(90∶10、70∶30、40∶60, v/v)对氯酸盐与高氯酸盐色谱行为的影响。结果如图1所示:随着有机相比例的增大,氯酸盐和高氯酸盐的保留时间缩短,同时峰宽变小、丰度逐渐增强;当水相与有机相比例为40∶60时,氯酸盐与高氯酸盐的色谱峰形尖锐,峰宽较小且对称度高。鉴于此,本研究选择0.1 mol/L乙酸铵水溶液-乙腈(40∶60, v/v)作为目标分析物的流动相。

**图1:**
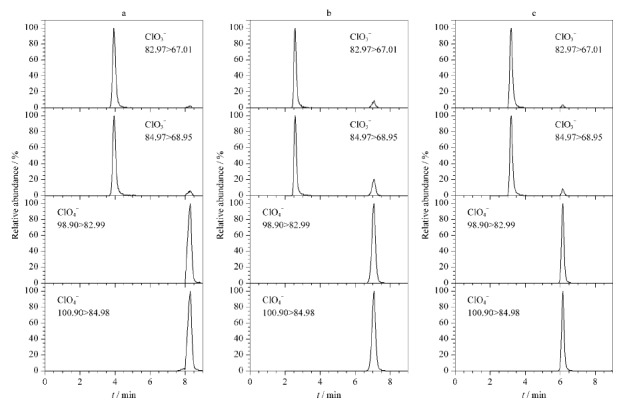
氯酸盐和高氯酸盐(100 μg/L)的提取离子色谱图

### 2.3 线性范围和检出限

在选定的最佳条件下,以氯酸盐和高氯酸盐的质量浓度为横坐标,其峰面积与内标物峰面积之比为纵坐标建立标准曲线,采用内标法进行定量。如表3所示,氯酸盐和高氯酸盐在2.00~200 μg/L与1.00~100 μg/L范围内线性关系良好,相关系数*r*^2^>0.9990。以目标分析物3倍信噪比(*S/N*)和10倍*S/N*计算方法的检出限(LOD)和定量限(LOQ),结果表明,氯酸盐与高氯酸盐的检出限分别为12.0 μg/kg和8.0 μg/kg,定量限分别为40.0 μg/kg和26.6 μg/kg。

**表3:** 目标分析物的线性方程、相关系数、线性范围、检出限与定量限(*n*=6)

Analyte	Linear equation	*r*^2^	Linear range/(μg/L)	LOD/(μg/kg)	LOQ/(μg/kg)
Cl ${O_{3}}^{-}$	*Y*=0.8199*X*+0.00623	0.9996	2.00-200	12.0	40.0
Cl ${O_{4}}^{-}$	*Y*=1.162*X*+0.00359	0.9998	1.00-100	8.0	26.6

*Y*: peak area ratio of analyte to IS; *X*: mass concentration, μg/L.

### 2.4 实际样品分析

采用本方法对市售的品牌茶叶样品(共计15份)进行测定。结果表明:市售茶叶中均未检出氯酸盐和高氯酸盐。以不含待测物的茶叶样品为空白,氯酸盐的加标水平为80、800和3200 μg/kg,高氯酸盐的加标水平为40、400和1600 μg/kg,平行测定7次,考察其回收率和重复性。由表4可知,氯酸盐和高氯酸盐的加标回收率为88.54%~97.25%,相对标准偏差(RSD)为3.2~5.2%。空白茶叶样品和加标样品的提取离子色谱图如图2所示。

**表4:** 目标分析物的加标回收率和精密度(*n*=7)

Analyte	Spiked level/(μg/kg)	Recovery/%	RSD/%
Cl ${O_{3}}^{-}$	80	89.56	4.2
	800	95.58	3.5
	3200	92.68	5.2
Cl ${O_{4}}^{-}$	40	88.54	4.9
	400	97.25	3.2
	1600	95.61	3.9

**图2:**
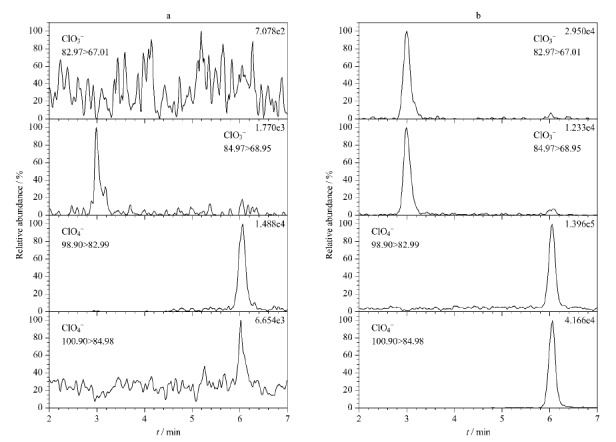
(a)实际样品和(b)加标样品中氯酸盐和高氯酸盐的提取离子色谱图

## 3 结论

本研究建立了茶叶样品中残留高氯酸盐与氯酸盐的离子交换色谱-串联质谱分析方法。该方法采用PRiME HLB固相萃取小柱对茶叶样品提取液进行分离净化,具有操作简单快速、准确度高、净化效果好等优势,适用于茶叶中氯酸盐和高氯酸盐含量的快速检测和定量分析。
